# Modeling and Solution of Signal Oscillation Mechanism of the Multi-Coil Sensor

**DOI:** 10.3390/s19163563

**Published:** 2019-08-15

**Authors:** Jiangbo Huang, Haowen Wang, Zhihong Fu, Wei Fu

**Affiliations:** 1 School of Electrical Engineering, Chongqing University, Chongqing 400044, China; 2 School of Robot Engineering, Yangtze Normal University, Chongqing 408100, China; 3 Operation and maintenance department, State Grid Chongqing Electric Power Company, Chongqing 400010, China

**Keywords:** multi-coil, sensor, signal oscillation

## Abstract

The multi-coil sensor consisting of a series of sub-coils provides a reliable way to avoid signal distortion from excitation field. Compared with conventional coil sensors, the multi-coil sensor exhibits more complex signal conversion performance, and the conventional equivalent circuit cannot reveal the possible attenuated oscillation, which seriously degrades the detection reliability. Based on a novel equivalent circuit model, this research investigates the causes of signal oscillation and proposes and validates an effective solution, which contributes to the signal transmission characteristics of multi-coil sensors for engineering applications.

## 1. Introduction

Multi-coil is an effective tool for detecting electromagnetic fields. Magnetic sensors based on air-core coils are widely used as a transducer in transient electromagnetic (TEM) exploration [1,2,3,4,5,6] and metallic crack detection [7,8,9,10], and coil sensors with integrated excitation module are commonly used in aeronautical transient electromagnetic detection [11], mine transient electromagnetic detection, and small loop towed systems [12].

**Figure 1:**
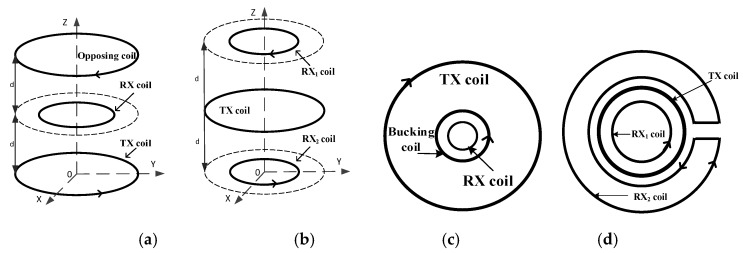
Schematic diagram of four multi-coil designs. (**a**) The opposing design. (**b**) The gradient design. (**c**) The bucking design. (**d**) The cross-loop design.

To avoid signal distortion caused by the excitation mixing phenomenon, the multi-coil designs that can effectively reduce the mutual inductance of the transmitter coil (TX coil) and the receiver coil (RX coil) are widely concerned. For example, the opposing design [13] and the gradient design respectively use two reverse-series sub-coils as transmitter or receiver modules, as shown in Figure 1a,b. Another coil design consists of non-uniform sub-coils, such as the bucking design [14] and cross-loop design [15] shown in Figure 1c,d, in which the cross-loop design adopting the forward-series mode has obvious advantages in detection sensitivity.

**Figure 2:**
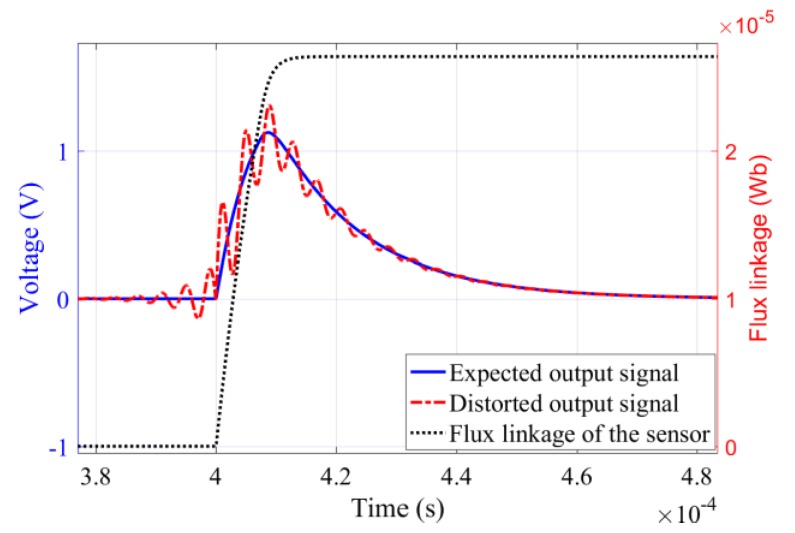
Attenuated oscillation of output signal.

Due to the miniaturization of coil sensors, the spacing of its sub-coils is significantly compressed [16]. However, in practice, we observed that the miniaturized cross-loop design may superimpose an attenuated oscillation in the non-periodic magnetic field response, as shown by the red dotted line in Figure 2. This signal oscillation seriously degrades the detection reliability [17], so it is necessary to analyze the signal mechanism of the multi-coil sensor to solve this problem.

## 2. Signal Transfer Features of General Coil Sensors

Based on the equivalent circuit model of the coil sensor, this section studies its signal transfer features under normal conditions.

**Figure 3:**
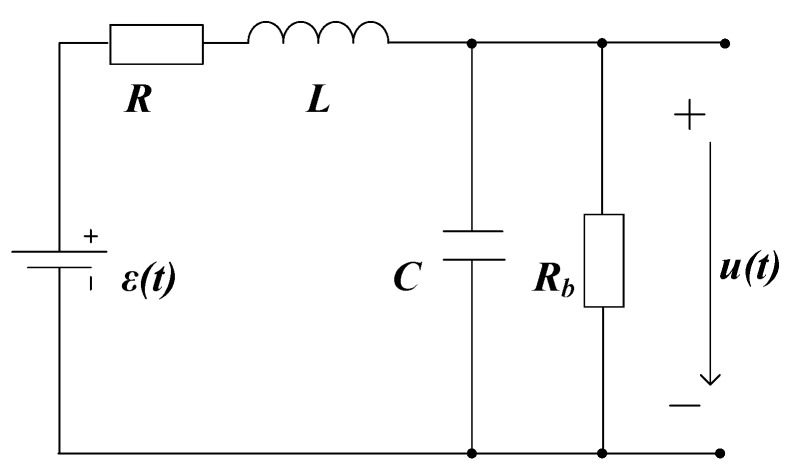
Equivalent circuit model of the receiver coil.

Figure 3 depicts the equivalent circuit of a general air-core coil. ε(t) denotes the induced electromotive force (EMF) of the coil, and u(t) is the output signal. Parameters L, R, and *C* are the inductance, the internal resistance, and the parasitic capacitance of the coil, respectively, $R_{b}$ is the damping resistance connected in parallel with the coil. Assume that no initial energy is stored in L and *C*. Then, ε(s) and U(s) in the s-domain can be related by the transfer function:

(1) $$H\left(s\right)=\frac{U\left(s\right)}{\varepsilon \left(s\right)}=\frac{1}{s^{2}LC+s\left(\frac{L}{R_{b}}+RC\right)+\frac{R+R_{b}}{R_{b}}}.$$

Equation (1) can be transformed into:

(2) $$\frac{\varepsilon \left(t\right)}{LC}=\frac{d^{2}u\left(t\right)}{dt^{2}}+2\delta_{1}\frac{du\left(t\right)}{dt}+\omega_{p}^{2}u\left(t\right)$$

where $\delta_{1}=\frac{1}{2}\left(\frac{R}{L}+\frac{1}{R_{b}C}\right)$, and $\omega_{p}=\sqrt{\frac{1}{LC}\left(\frac{R}{R_{b}}+1\right)}$ is the coil resonance frequency. Define the damping coefficient

(3) $$\xi =\frac{\delta_{1}}{\omega_{p}}=\frac{R_{b}RC+L}{2\sqrt{LCR_{b}\left(R+R_{b}\right)}}.$$

**Figure 4:**
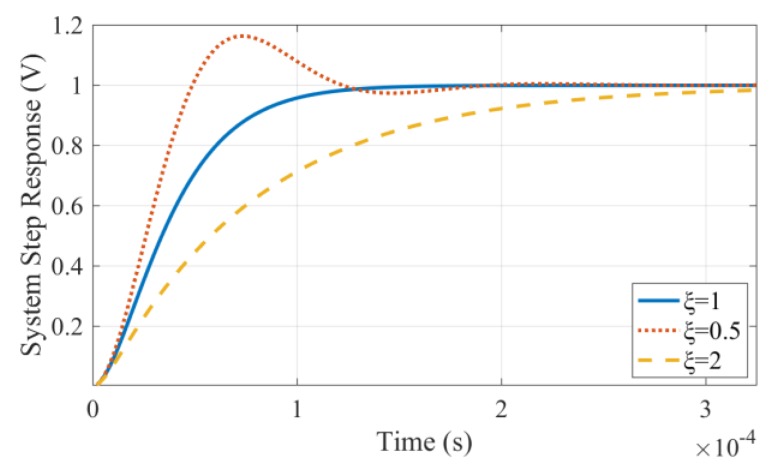
Comparison of the step response of the coil sensor under different damping conditions.

In the case of ξ  = 1, the coil works under the critical damping state and its unit step response is shown by the solid blue line in Figure 4; in the underdamped state ξ  < 1, the unit step response oscillates, as shown by the red dotted line in Figure 4; in the over-damped state ξ  > 1, the response attenuation is slow, as shown by the yellow dotted line in Figure 4 [18].

Under the condition of critical damping ξ = 1, we obtain the critical damping resistance

(4) $$R_{bd}=\frac{L}{RC+2\sqrt{LC}}.$$

In general, a damping resistance with a value less than $R_{bd}$ can avoid oscillation for the coil sensor. However, the multi-coil sensor is possible to output a non-periodic magnetic field signal in the form of attenuated oscillations, as shown by the red dotted line in Figure 2, even if the damping resistor is in the overdamped range. Therefore, the cause of signal oscillation needs to be found to ensure sensor reliability.

## 3. Modeling of Multi-Coil Sensor

In general, if an evenly arranged coil is arbitrarily divided into two parts, their parameters satisfy $\frac{R_{1}}{R_{2}}=\frac{L_{1}}{L_{2}}\approx \frac{C_{2}}{C_{1}}$, where $R_{1}$, $R_{2}$, $L_{1}$,$\;L_{2}$, $C_{1}$, and $C_{2}$ respectively represent the internal resistance, inductance, and parasitic capacitance of the two sub-coils. Here, coils satisfying the above condition are marked as the matching coil system, which generally provides a stable signal transmission, such as the two sub-receiver coils of the gradient design. However, for the non-matching receiver coils of the bucking design or the cross-loop design, the signal oscillation is often triggered when the sub-coils are closely distributed or there are good conductors near the coils. Therefore, we speculate that this signal oscillation phenomenon is related to the parasitic capacitance between non-matching sub-coils.

To verify this conjecture, we explore the effect of the matching state and parasitic capacitance distribution of the sub-coils on the signal oscillation, and try to find an equivalent circuit model, which can simulate the signal oscillation properly.

**Figure 5:**
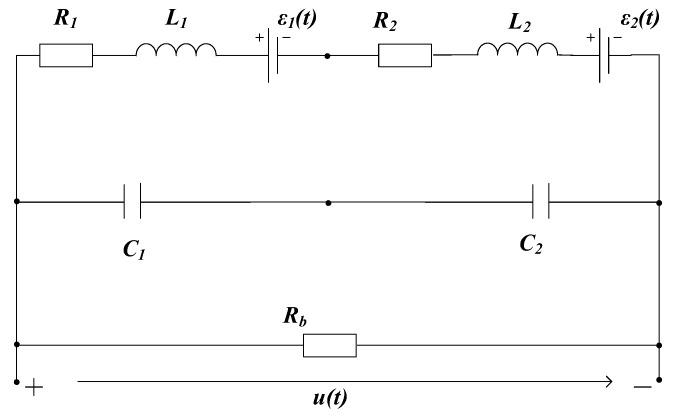
Model 1: Equivalent circuit with a single damper resistance.

The equivalent circuit model 1, which is composed of two sub-coils simply connected in a series, is established, as shown in Figure 5. The internal resistance and inductance of the two sub-coils are connected in series in the same branch. The equivalent lumped capacitances of the two sub-coils are connected in series in the other branch, and the two branches are in parallel with the damping resistor $R_{b}$. Assuming i(t) is the induced eddy current of the measured object, the coupling coefficients of the eddy current source and the two sub-coils are denoted as $M_{1}$ and $M_{2}$, respectively; thus, the EMF of the multi-coil sensor system can be solved by

(5) $$\varepsilon \left(t\right)=\varepsilon_{1}\left(t\right)+\varepsilon_{2}\left(t\right)=-(M_{1}+M_{2})\frac{di\left(t\right)}{dt}.$$

Setting parameters of a matching coil system: $R_{1}=R_{2}=8\;\Omega$, $L_{1}=L_{2}=60\;\mathrm{mH}$, $C_{1}=C_{2}=0.5\;\mu F$, $R_{b}=1.02\;k\Omega$. Setting parameters of a non-matching coil system: $R_{1}=8\;\Omega$, $L_{1}=60\;\mathrm{mH}$, $C_{1}=0.5\;\mu F$, while $R_{2}=32\;\Omega$, $L_{2}=960\;\mathrm{mH}$, $C_{2}=2\;\mu F$, and the damping resistance $R_{b}=1.02\;k\Omega$. The values of both damping resistance $R_{b}$ ensure that model 1 is in an overdamped state. 

**Figure 6:**
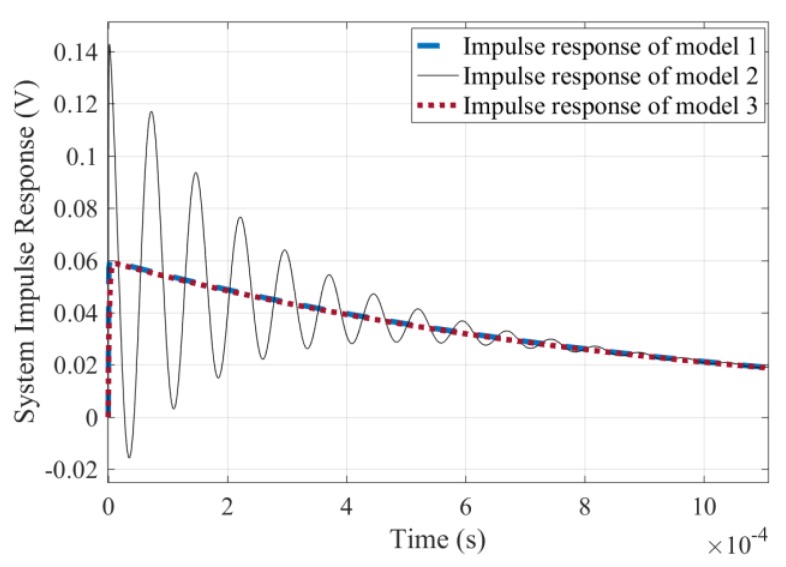
Impulse response simulation signals of three models.

Taking $\frac{di\left(t\right)}{dt}$ as the system input and u(t) as the system output, the unit impulse response of model 1 does not oscillate under the two parameter samples regardless of the value of $M_{1}$ and $M_{2}$, as shown by the blue dashed line in Figure 6 in the case of $M_{1}=1\times 10^{-5}$, $M_{2}=5\times 10^{-5}$, i(t<0)=0, and i(t≥0)=1. Therefore, model 1 cannot be used to simulate the signal oscillation.

**Figure 7:**
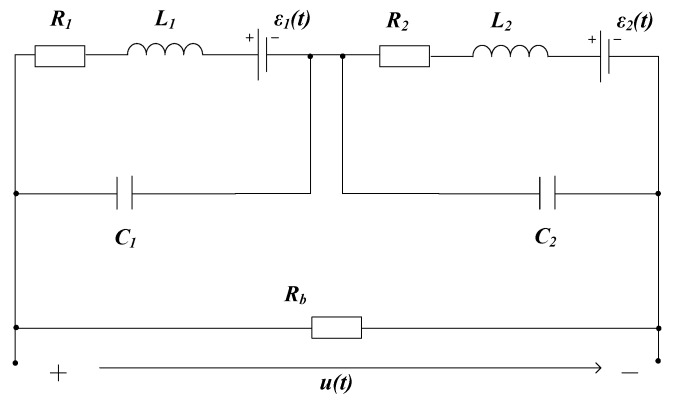
Model 2: Equivalent circuit model with a single damper resistance.

Next, a novel equivalent circuit model 2 shown in Figure 7 is established, in which the equivalent lumped capacitances of the two sub-coils are connected in parallel to the respective inductance branches. Regardless of the value of $M_{1}$ and $M_{2}$, the unit impulse response of Model 2 does not oscillate for the matching coil system. However, the signal oscillation phenomenon cannot be masked under the non-matching coil system, as shown by the solid black line in Figure 6 in the case of $M_{1}=1\times 10^{-5}$ and $M_{2}=5\times 10^{-5}$. Therefore, model 2 successfully simulated the signal oscillation phenomenon under the non-matching parameters case.

For the multi-coil system, it can be seen from the above analysis that the non-matching parameters of the sub-coils and the discontinuous arrangement of the parasitic capacitance are the key factors in signal oscillation.

## 4. Solution for Signal Oscillation

**Figure 8:**
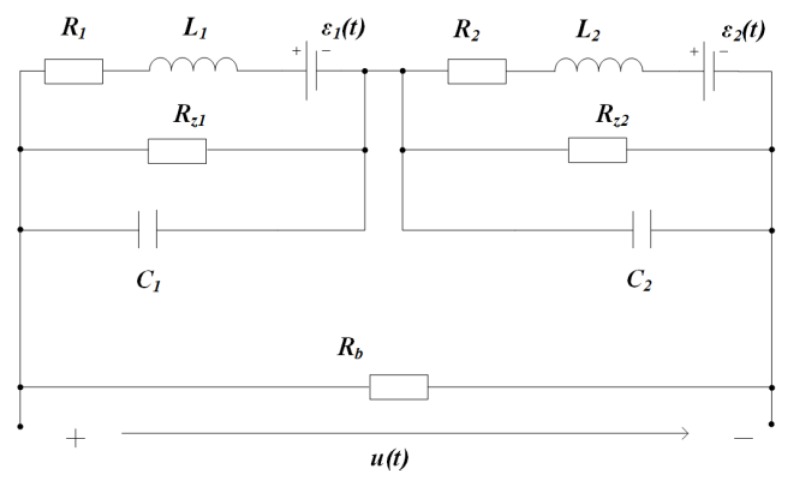
Model 3: Dual-damping resistance equivalent circuit.

To eliminate the signal oscillation phenomenon of the non-matching coil system, we propose the sub-damping solution: Set a damping resistor at each sub-coil port, and the ratio of each sub-damper is consistent with that of the corresponding inductance coefficients, that is, $\frac{R_{z1}}{R_{z2}}=\frac{L_{1}}{L_{2}}$. With this scheme, a double-damping equivalent circuit is established in Figure 8, which is marked as model 3. The parameters of the two sub-coils are kept the same as those of the equivalent circuit model 2, and the matching sub-damping resistors: $R_{z1}=60\;\Omega$, $R_{z2}=960\;\Omega$, while the total port resistance is taken as $R_{b}=100\;k\Omega$. In this case, the decay oscillation of the unit impulse response is eliminated, as shown by the red dotted line in Figure 6.

It can be seen from Figure 6 that the circuit model 2 can simulate the signal oscillation of non-matching coil systems and adding the sub-damping resistor to each sub-coil is an effective scheme to eliminate the oscillation phenomenon, which is called the sub-damping solution.

## 5. Simulation

Based on the transfer function of the multi-coil sensor, this section analyzes the mechanism of the sub-damping solution.

Taking $\frac{di\left(t\right)}{dt}$ as the system input and u(t) as the system output, the transfer function of model 2 is:

(6) $$H_{1}\left(s\right)=-\frac{f_{1}s^{2}+g_{1}s+h_{1}}{a_{1}s^{4}+b_{1}s^{3}+c_{1}s^{2}+d_{1}s+e_{1}}.$$

where

$a_{1}=L_{1}L_{2}C_{1}C_{2}R_{b}$;

$b_{1}=L_{1}C_{1}C_{2}R_{2}R_{b}+L_{2}C_{1}C_{2}R_{1}R_{b}+L_{1}L_{2}C_{1}+L_{1}L_{2}C_{2}$;

$c_{1}=L_{1}C_{1}R_{2}+L_{1}C_{2}R_{2}+L_{2}C_{1}R_{1}+L_{2}C_{2}R_{1}+C_{1}C_{2}R_{1}R_{2}R_{b}+L_{1}C_{1}R_{b}+L_{2}C_{2}R_{b}$;

$d_{1}=L_{1}+L_{2}+C_{1}R_{1}R_{2}+C_{2}R_{1}R_{2}+C_{1}R_{1}R_{b}+C_{2}R_{2}R_{b}$;

$e_{1}=R_{1}+R_{2}+R_{b}$;

$f_{1}=L_{2}C_{2}R_{b}M_{1}+L_{1}C_{1}R_{b}M_{2}$;

$g_{1}=C_{2}R_{2}R_{b}M_{1}+C_{1}R_{1}R_{b}M_{2}$;

$h_{1}=R_{b}(M_{1}+M_{2})$.

**Figure 9:**
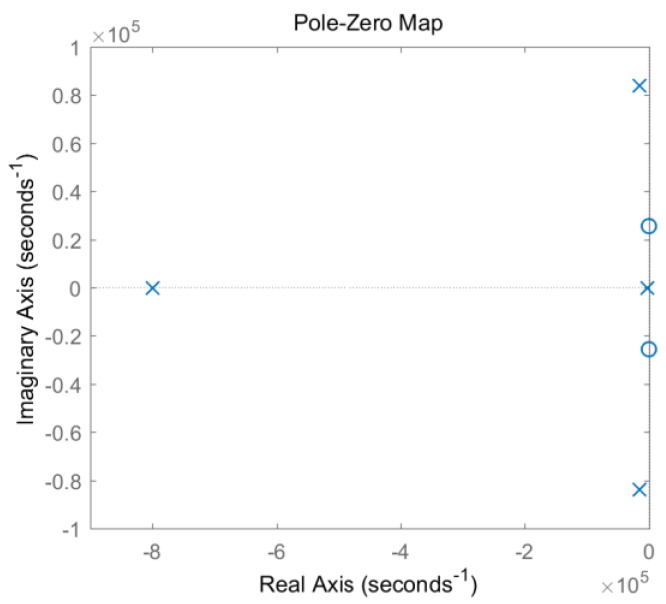
Zero-pole diagram of circuitry model 2.

According to the relationship between system stability and zero-pole distribution [19], if all system poles are on the real axis, the system is an over-damped system, and its unit impulse response is a non-periodic waveform. When the system has a conjugate complex pole, its unit impulse response exhibits a damped oscillation process. The pole-zero diagram of model 2 under the non-matching parameter is plotted in Figure 9. Due to the existence of the conjugate complex pole, the signal must have a damped oscillation as shown by the red dotted line in Figure 6.

Similarly, the transfer function of model 3 is:

(7) $$H_{1}\left(s\right)=-\frac{f_{1}s^{2}+g_{1}s+h_{1}}{a_{1}s^{4}+b_{1}s^{3}+c_{1}s^{2}+d_{1}s+e_{1}}.$$

It also takes $\frac{di\left(t\right)}{dt}$ as the system input and u(t) as the system output, where

$a_{2}=L_{1}L_{2}C_{1}C_{2}R_{z1}R_{z2}R_{b}$;

$b_{2}=L_{1}L_{2}C_{1}R_{z1}R_{z2}+L_{1}L_{2}C_{2}R_{z1}R_{z2}+L_{1}L_{2}C_{1}R_{z1}R_{b}+L_{1}L_{2}C_{2}R_{z2}R_{b}+L_{1}C_{1}R_{2}R_{z1}R_{z2}R_{b}$$+L_{2}C_{1}R_{1}R_{z1}R_{z2}R_{b}$;

$c_{2}=L_{1}C_{1}R_{z1}R_{z2}R_{b}+L_{2}C_{2}R_{z1}R_{z2}R_{b}+L_{1}C_{2}R_{z1}+L_{1}C_{2}R_{z2}+L_{1}C_{2}R_{2}R_{z1}R_{z2}+L_{2}C_{1}R_{1}R_{z1}R_{z2}+$$L_{1}C_{2}R_{2}R_{z2}R_{b}+L_{2}C_{1}R_{1}R_{z1}R_{b}+L_{1}L_{2}R_{b}+L_{1}C_{1}R_{z1}R_{z2}R_{2}+L_{2}C_{2}R_{z1}R_{z2}R_{1}+L_{1}C_{1}R_{z1}R_{b}R_{2}$$+L_{2}C_{2}R_{b}R_{z2}R_{1}+C_{1}C_{2}R_{1}R_{2}R_{z1}R_{z2}R_{b}$;

$d_{2}=L_{2}R_{z1}R_{z2}+L_{1}R_{z1}R_{z2}+L_{2}R_{z1}R_{b}+L_{1}R_{2}R_{b}+C_{2}R_{2}R_{z1}R_{z2}R_{b}+C_{1}R_{1}R_{z1}R_{z2}R_{b}+L_{1}R_{2}R_{z1}+$$L_{2}R_{1}R_{z2}+L_{1}R_{2}R_{z2}+L_{2}R_{1}R_{z1}+L_{1}R_{2}R_{b}+L_{2}R_{1}R_{b}+C_{2}R_{1}R_{2}R_{z1}R_{z2}+C_{1}R_{1}R_{2}R_{z1}R_{z2}$$+C_{2}R_{1}R_{2}R_{z2}R_{b}+C_{1}R_{1}R_{2}R_{z1}R_{b}$;

$e_{2}=R_{2}R_{z1}R_{b}+R_{1}R_{z2}R_{b}+R_{z1}R_{z2}R_{b}+R_{2}R_{z1}R_{z2}+R_{1}R_{z1}R_{z2}+R_{1}R_{2}R_{z1}+R_{1}R_{2}R_{z2}+R_{1}R_{2}R_{b}$;

$f_{2}=L_{2}C_{2}R_{z1}R_{z2}R_{b}M_{1}+L_{1}C_{1}R_{z1}R_{z2}R_{b}M_{2}$;

$g_{2}=\left(C_{2}R_{2}R_{z1}R_{z2}R_{b}+L_{2}R_{z1}R_{b}\right)M_{1}+\left(C_{1}R_{1}R_{z1}R_{z2}R_{b}+L_{1}R_{z2}R_{b}\right)M_{2}$;

$h_{2}=\left(R_{2}R_{z1}R_{b}+R_{z1}R_{z2}R_{b}\right)M_{1}+\left(R_{1}R_{z2}R_{b}+R_{z1}R_{z2}R_{b}\right)M_{2}$.

**Figure 10:**
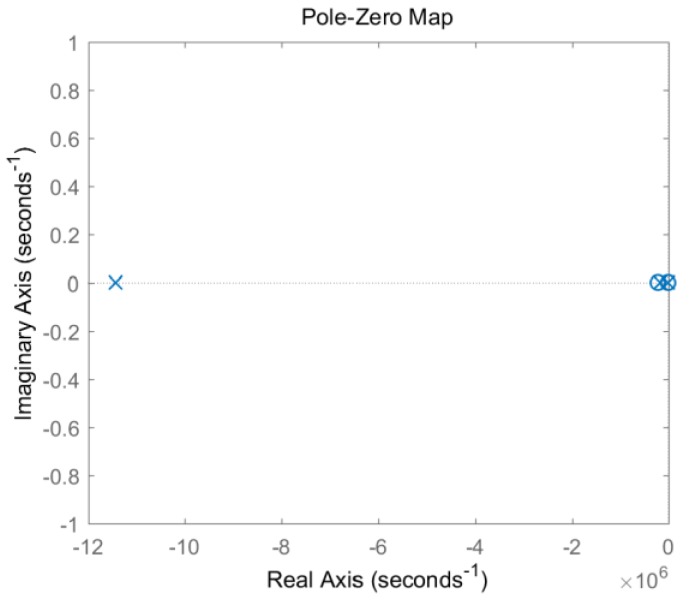
Zero-pole diagram of double-damped circuit model 3.

In the case of $R_{z1}=60\;\Omega$, $R_{z2}=960\;\Omega$, $R_{b}=100\;k\Omega$, the pole-zero diagram of model 3 is shown in Figure 10. It can be seen that all poles are moved to the negative real axis, thus the system enters an overdamped state, and thus avoids the signal oscillations.

**Figure 11:**
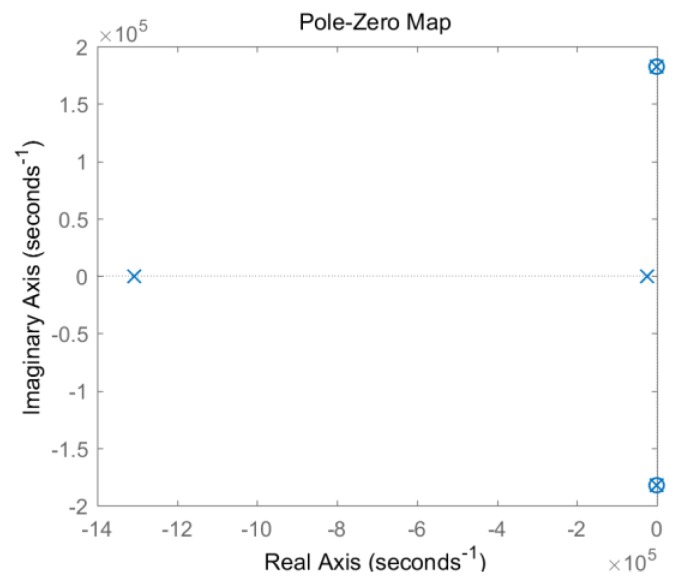
Zero-pole diagram of symmetrical circuit model 2.

For the matching parameter system, the conjugate pole based on circuit model 2 is cancelled by the zero point, as shown in Figure 11, thereby avoiding signal oscillation.

## 6. Comparison Test

This section examines the effect of the sub-damping solution on the signal oscillation phenomenon by a comparative experiment.

**Figure 12:**
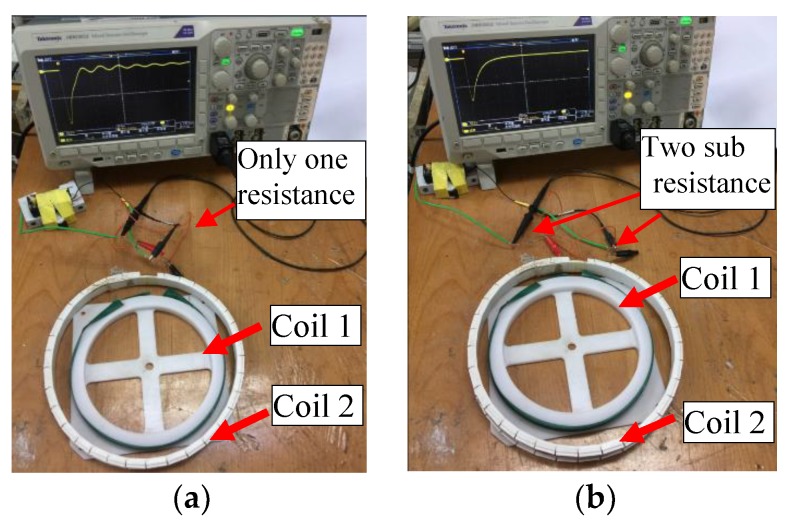
Comparison of two multi-coil systems. (**a**) Single-damped coil system; (**b**) double-damped coil system.

**Figure 13:**
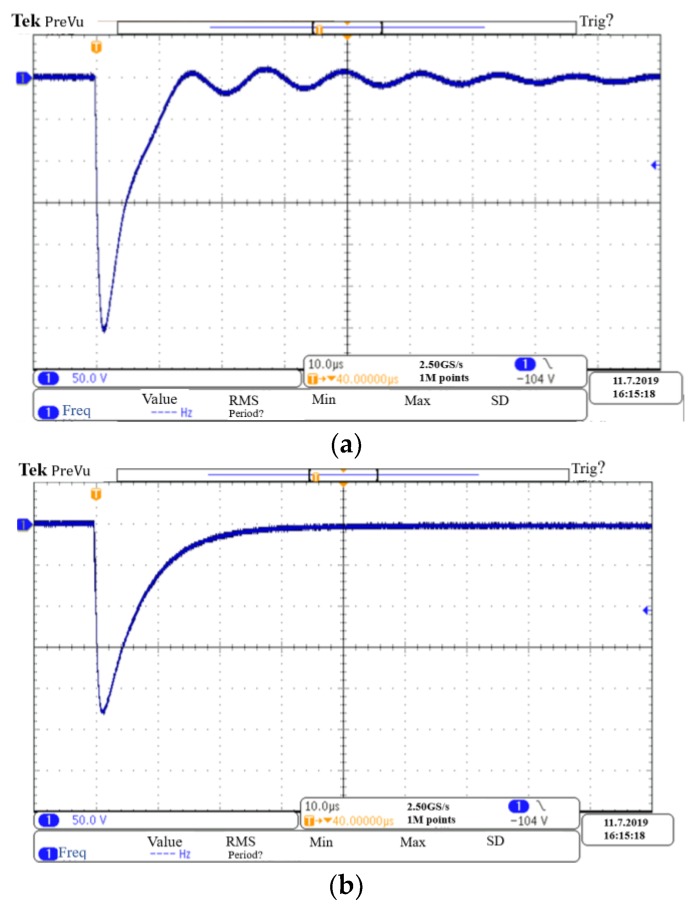
Impulse response waveforms of two multi-coil systems. (**a**) Single-damped coil system; (**b**) double-damped coil system.

Two multi-coil systems are built with non-matching sub-coils: One 300-turn coil with a diameter of 220 mm is marked as Coil 1, and the other 150-turn coil with a diameter of 200 mm is marked as Coil 2. In the first coil system, the output port of Coil 1 is directly connected in series with the input port of Coil 2, and only one resistor is arranged at the ports of the multi-coil sensor to form a single-damped coil system in an overdamped state, as shown in Figure 12a. Another coil system adopted the sub-damping solution, thus sub-damper resistors matching the corresponding sub-coil inductance are respectively added to the ports of Coil 1 and Coil 2 to form a double-damped coil system, as shown in Figure 12b. The two sub-coils are separated by less than 12 mm. When the DC voltage source connected in parallel to the total ports of each coil system is quickly cut off, the corresponding impulse response waveforms that caused by the zero-input response are as shown in the oscilloscope [20], which can be used as an indicator of system stability. It is known from Figure 13b that the double-damper coil system avoids the oscillation phenomenon that occurs in the single-damper coil system, which is shown in Figure 13a. Therefore, the sub-damping solution proposed in this paper can effectively solve the signal oscillation problem of the multi-coil system.

## 7. Conclusions

In the case where the sub-coils pitch is too small, the multi-coil sensor with non-matching parameters has a pair of conjugate complex poles, which is the main cause of the signal oscillation. There are three ways to solve this problem: One is to select the sub-coils with matching parameters, but it is difficult to get matching sub-coils for the bucking design and the cross-loop design shown in Figure 1; another method is to increase the spacing between sub-coils to balance the distribution state of the parasitic capacitance, so that signal oscillation is avoided, but this requires a fairly high wiring technology of the miniaturized coil sensor; the third solution is to set a sub-damping resistor for each sub-coil, as shown in Figure 8; the sub-damping resistor acts to move the original conjugate complex pole to its real-axis, thus eliminating the signal oscillation. The third solution, named the sub-damping solution, is universally applicable, which provides a theoretical basis for the study of the signal transmission features of the multi-coil sensor and its calibration work.
